# Perspectives from oncology patient navigation programs on information management practices and needs: a descriptive study

**DOI:** 10.1007/s00520-019-04837-7

**Published:** 2019-05-09

**Authors:** Serena Phillips, Sarah Raskin, Yuqing Zhang, Mandi Pratt-Chapman

**Affiliations:** 1 Institute for Patient-Centered Initiatives and Health Equity, The George Washington University Cancer Center, 2600 Virginia Avenue NW, Suite 300, Washington, DC 20037 USA; 2 L. Douglas Wilder School of Government and Public Affairs, Virginia Commonwealth University, 923 W. Franklin Street, Richmond, VA 23284 USA

**Keywords:** Patient navigation, Health information technology, Oncology, Qualitative

## Abstract

**Purpose:**

The purposes of this study are to describe oncology patient navigation (PN) program perspectives on: (1) use of information systems and processes, (2) uses of program data, and (3) desired information system characteristics.

**Methods:**

We conducted multi-phase data collection to inform development of the Patient Navigation Barriers and Outcomes Tool™ (PN-BOT™), a new information management and reporting tool for oncology PN programs. Phase I was a national online survey of PN staff (*n* = 343) about data practices. Phase II was a pilot test of a PN-BOT™ prototype with nine PN programs. Survey results were tabulated. Qualitative analysis identified emergent themes from open-response fields from the Phase I survey and open-response survey and interview data from Phase II pilot testers.

**Results:**

PN program information management practices and systems were diverse and often leveraged a patchwork of untailored platforms. Navigators used data to inform navigation tasks, service improvement, research, and reporting. Respondents desired a streamlined, integrated, affordable data system able to minimize data entry burden, meet needs of diverse stakeholders, facilitate navigation work, readily generate reports, and share information among healthcare team members.

**Conclusions:**

Although oncology navigation programs explore diverse solutions, programs struggle to find health information technologies that sufficiently meet their needs. Information systems designed for oncology PN programs should perform a wide range of functions: be customizable, affordable, interoperable, and have low data entry burden. Organizations exploring solutions should invite PN input in decisions. PN-BOT™ was developed as a free Excel-based tool for PN programs responsive to reported needs.

## Background

Oncology patient navigation (PN) programs have proliferated rapidly throughout the USA as a promising approach to achieving health equity in cancer care [1–3]. PNs help coordinate care and remove barriers to care [4]. Researchers and practitioners have recognized the importance of robust data and metrics for PN program evaluation, especially as a relatively new field with diverse mechanisms of funding and sustainability [5–11]. Capacity to capture high-quality data has been a challenge. The great diversity of PN programs, lack of standardized metrics, and varying definitions of concepts have made cross-program comparisons and generalization of outcomes difficult [5, 11–14]. Recommendations to improve data quality and rigor have included: detailed reporting of program characteristics, prioritization of common standardized outcome metrics, use of validated patient-reported outcomes measures, and shared databases across institutions [5, 12, 14].

Information systems and processes are important to PN program functioning and evaluation. They systematically capture, track, and synthesize information about patient characteristics, navigation interventions, important dates, follow-up needed, and outcomes achieved [15, 16]. In a qualitative study, nurse practitioner PNs reported use of tracking systems during all phases of care as a means of interfacing with both patients and the larger care system [15]. A variety of options exist. Navigators have reported using chart review templates, spreadsheets, sticky notes, Outlook alerts, data specialists, both formal and homegrown software programs, and other processes [15]. Meanwhile, Ajeesh and Luis described a PN module embedded in an electronic health record (EHR) for colorectal cancer navigation, which had a screening registry, navigation tracking, and educational tools for data collection, retrieval, and support of patient decision-making [17].

Despite the importance to PN research and evaluation, there has been little targeted assessment of PN information system practices, needs, and barriers. The technology acceptance model theorizes that perceived usefulness and ease of use predict attitudes, behavioral intentions, and actual use of information systems [18]. Thus, PN perspectives are critical to understand. We conducted secondary analysis on data previously collected through a national PN survey and interviews with PN staff in order to obtain perspectives on the following descriptive research questions: (1) What is the landscape of PN information management systems and processes? (2) For what purposes do PN programs use data? (3) What information system characteristics are important to navigation programs?

## Methods

### Study design

**Fig. 1:**
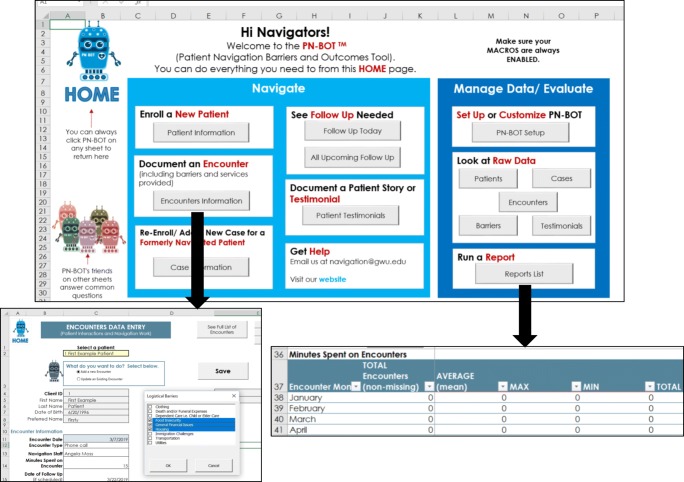
Patient Navigation Barriers and Outcomes Tool (PN-BOT™) user interface examples: home menu, data entry form, and automatic report; downloadable at http://bit.ly/AboutPNBOT

**Fig. 2:**
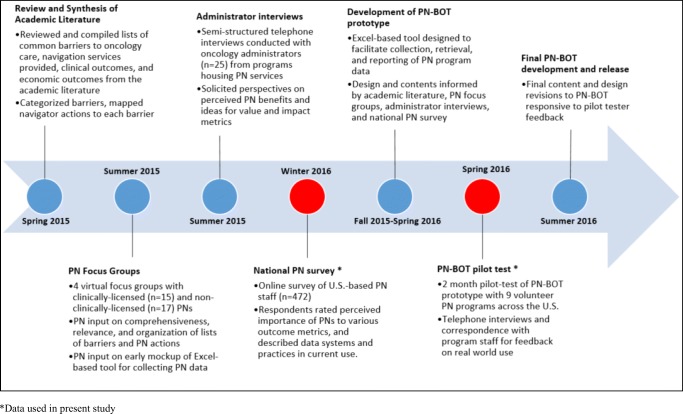
Timeline of multi-phase data collection activities informing development of the Patient Navigation Barriers and Outcomes Tool (PN-BOT)

Data used in this study were originally collected to inform development of a new, cost-free, data management and evaluation tool designed for oncology PN programs called the “Patient Navigation Barriers and Outcomes Tool™,” or “PN-BOT™” (Fig. 1) [19]. PN-BOT™ is a macro-enabled Microsoft Excel workbook that allows PN programs to enter, retrieve, and automatically generate reports on client data. Variable fields are customizable, with the option to capture information on client demographics, contact information, cancer and treatment details, navigator time spent, barriers to care, services provided, and outcomes. To maximize PN-BOT™’s responsiveness to PN data needs, we engaged in a year-long multi-phase formative research process detailed in Fig. 2.

PN-BOT™ development is not the focus of this study; however, two phases in its development process lent relevant insights to present research aims: (1) a national survey of PN programs in January 2016, and (2) a pilot test of a PN-BOT™ prototype to assess real world use from February to May 2016. Research study activities were reviewed by the GW Office of Human Research (protocol #051510), designated exempt, and approved.

### Phase I: national survey

A survey about PN outcome metrics and data practices was administered using Research Electronic Data Capture (REDCap), a secure web-based platform [20]. A convenience sample of PN staff was recruited through professional listservs, GW Cancer Center social media postings, professional organization message boards, and email correspondence with personal contacts. Individuals were eligible to participate if they (1) identified as a navigator of any type or as someone with a managing, supervising, or evaluating role in a PN program; and (2) worked with a US-based PN program primarily providing cancer services. Informed consent was obtained from all individual participants included in the study. As an incentive, respondents were offered entry into a drawing for $1000 towards professional development. Out of 472 eligible respondents who started the survey, 343 completed the final section on data practices, containing the multiple-choice question: “How do you currently collect, document, and track information needed to provide patient navigation services?” A total of 111 respondents provided a text response describing their current or preferred methods of data management or gave comments in response to the prompt, “Do you have any additional thoughts or explanations to share regarding how best to document the value of navigation at your institution?”

### Phase II: PN-BOT™ pilot test

Representatives from nine PN programs across the USA volunteered to participate in a 2-month pilot test of the PN-BOT™ prototype. Participants were recruited from those expressing interest in re-contact in previous data collection phases and individuals who initiated contact with GW to request PN evaluation-related technical assistance. Prior to the pilot, participants completed a web-based background survey. In open-response questions, pilot test participants were asked to describe: (1) information systems and procedures currently in place for documentation and tracking, (2) uses of data collected, (3) planned utilization of PN-BOT™, and (4) additional comments. After at least 1 week of using the PN-BOT™ prototype, one group telephone interview was conducted per program. All PN staff at each site were welcome to participate. Each semi-structured interview typically lasted 40–60 min and included one to five PN staff from the same program (total *n* = 23). Two interviews were conducted with Pilot Site B to accommodate staff scheduling conflicts, for a total of 10 interviews. Most pertinent questions included: “What information is most important for your program to track and document?”, “What kinds of reports does your program hope to generate?”, and questions about anticipated barriers and benefits to PN-BOT™ use. Pilot test interviews were audio recorded and transcribed verbatim.

### Data analysis

Descriptive statistics for survey participants were obtained using Stata 13. Secondary analysis of qualitative data began with identifying sections of the national survey, pilot background survey, and pilot interview transcripts most relevant to the present research questions. Qualitative data from all sources were imported into NVivo 10 and coded together using an applied pragmatic approach, which Goldkuhl characterizes as “knowledge …used in action for making a purposeful difference in practice” [21]. A pragmatic approach prioritizes problem-solving and is not allied with any one philosophical orientation [22]. While the overarching research questions were determined a priori, themes were allowed to inductively emerge.

Coding was conducted by authors SP and YZ, research staff with backgrounds in public health. SP developed the preliminary codebook based on initial review of all data using open coding. YZ independently applied the preliminary codebook to a subset of responses, and discrepancies were discussed to revise the codebook. YZ and SP independently applied the finalized codebook, meeting regularly to discuss coding discrepancies until agreement was reached. MPC and SR (principal investigator and qualitative methods expert, respectively) provided input and reviewed the final codebook for conceptual soundness. The pilot provided a natural opportunity for reciprocity and validation of findings with participants. Memos were written to practice self-reflexivity. Use of multiple data sources allowed for triangulation [23].

## Results

### Participant characteristics

Phase I national survey respondent characteristics are detailed in Table 1 (*n* = 343). Respondents were overwhelmingly female (91.76%), non-Hispanic (83.58%), and White (82.22%). Roughly one quarter (26.82%) indicated a supervisory, managing, or evaluating primary role in their oncology navigation program (heretofore referred to as “administrative”). There was representation from 44 states and the District of Columbia.

**Table 1:** National PN survey participant characteristics (*n* = 343)

Characteristic	Frequency (%)
Navigation setting	
Hospital/cancer center/outpatient clinic	273 (79.59)
Non-profit organization	34 (9.91)
Other	36 (10.50)
Primary role	
Nurse navigator	139 (40.52)
Supervisor/program manager/evaluator	92 (26.82)
Patient navigator (non-clinical)	64 (18.66)
Community health worker	18 (5.25)
Volunteer/peer navigator (unpaid)	16 (4.66)
Clinical social worker	14 (4.08)
Navigator types in organization*	
Nurse navigators	230 (67.06)
Patient navigators (non-clinical)	142 (41.40)
Clinical social workers	111 (32.36)
Community health workers	53 (15.45)
Volunteer/peer navigators (unpaid)	40 (11.66)
Other	13 (3.79)
Geographic region	
South	151 (44.02)
West	80 (23.32)
Midwest	62 (18.08)
Northeast	50 (14.58)
Cancer continuum stage served*	
Outreach to get people into screening	167 (48.69)
Screening to diagnostic resolution	180 (52.48)
Diagnosis to treatment	292 (85.13)
Through treatment	266 (77.55)
Post-treatment survivorship	230 (67.06)
End of life/hospice or palliative	146 (42.57)
Cancer type*	
No cancer	43 (12.54)
Any or all cancer	207 (60.35)
Breast cancer	153 (44.61)
Lung cancer	88 (25.66)
Colorectal/anal cancer	76 (22.16)
Prostate cancer	64 (18.66)
Gynecological cancers	61 (17.78)
Other cancers	94 (27.41)
How do you currently collect, document, and track information needed to provide patient navigation services?*	
None	27 (7.87)
Case management software	30 (8.75)
Seamless use of electronic health record**	83 (24.20)
Limited use of electronic health record***	127 (37.03)
Microsoft Excel spreadsheet	161 (46.94)
Microsoft Access database	38 (11.08)
Paper forms: given to patients to self-report	55 (16.03)
Paper forms: completed by navigator	135 (39.36)
Other (specify)	33 (9.62)
Number of data collection methods in place	
0	27 (7.87)
1	109 (31.78)
2	105 (30.61)
3	75 (21.87)
4+	27 (7.87)

*Multiple selections permitted; categories are not mutually exclusive

**We defined “seamless use of Electronic Health Record” in the survey as: “Navigation-related documentation is integrated into the patient’s clinical records in the EHR; navigators can directly enter and retrieve information from the EHR”

***We defined “limited use of Electronic Health Record” in the survey as: “Navigation-related documentation is separate from the patient’s clinical records in the EHR; navigators may have some access to the EHR, but still need to manually copy out needed information”

Characteristics of Phase II PN-BOT™ pilot test PN programs (*n* = 9) are described in Table 2. Most were hospital or cancer center based (*n* = 7). The 23 interviewed PN staff were all female and included nurse navigators (*n* = 8), administrators (*n* = 6), non-clinical patient navigators (*n* = 5), and other clinically-licensed navigators (*n* = 4). Pilot sites described motivations for testing PN-BOT™ such as wishing to explore options for streamlining current data processes, increasing reporting rigor and capabilities, and demonstrating program value.

**Table 2:** Pilot PN Program characteristics (*n* = 9)

Pilot site	Navigation setting	Geographic region	PN type(s)*	Funding source(s)	Est. new patients per year per PN	Data and tracking mechanisms used for navigation services
A	Hospital/cancer center	Midwest	NN	Organizational budget	101–200	EHR, Excel
B	Hospital/cancer center	West	NN	Organizational budget	201–300	EHR, Excel, Lotus calendar, Cancer Support Community
C	Hospital/cancer center	South	NN, SW, FN, V, O	Organizational budget, grant or foundation funding	301–400	EHR, Excel
D	Hospital/cancer center	Northeast	PN, SW	Organizational budget	401–500	Access
E	Hospital/cancer center	Northeast	PN, NN, CHW, V	Organizational budget, grant or foundation funding	101–200	EHR, Excel
F	Non-profit	South	PN, SW, V	Grant or foundation funding, fundraising, philanthropic donations	301–400	Salesforce
G	Non-profit	West	PN	Organizational budget, grant or foundation funding, fundraising, philanthropic donations, in-kind services	101–200	Excel, Access
H	Hospital/cancer center	South	NN	Organizational budget	51–100	EHR, Excel
I	Hospital/cancer center	South	NN, SW, FN	Organizational budget	101–200	EHR, Excel

*NN, nurse navigator; *PN*, non-clinically licensed patient navigator; *SW*, social worker; *FN*, financial navigator; *CHW*, community health worker; V, unpaid volunteer/peer navigator; *O*, other

Supplemental quotes in Table 3 illustrate themes. Original spellings and punctuations are preserved in quotes unless otherwise noted.

**Table 3:** Quotations illustrating qualitative themes, Phase I national survey, and Phase II pilot background survey and interviews

Domains and themes	Quotes
PN information systems landscape	
Diverse data systems, multiple simultaneous use	“Currently the process for capturing and tracking all our metrics is a combination of piecemeal EHR and paper, and the tabulation and interpretation of the data is labor intensive.” (Pilot Site E)
Tailoring issues	“We do not have an electronic system specific for navigation and our hospital system uses multiple systems for various services.” (Admin, Phase I survey)*
Data system decisions in flux	“We just began using EPIC in the past few months. Prior to that used a paper process so we are between the 2 systems. We will be receiving the Beacon module by the end of the year.” (NN, Phase I Survey) “In the past we had a different EMR that allowed us to capture much more meaningful data on patient navigation due to the ability to build individual documentation templates which could then provide us with data by nurse, by type of encounter etc. We can no longer do that as a result of being part of a system with required standardization that does not always provide us with the same detail” (Admin, Phase I Survey) “We’ve been playing around with SharePoint to see if it can serve all of the purposes we need.” (Admin, Phase I Survey)
PN data uses	
Direct provision of navigation services	“we collect information in order to provide relevant educational materials, assist patients in finding doctors who have experience with this rare disease so they get the best treatment possible, connect patients with one another and other resources for support.” (Pilot Site F)
Service and care improvements	“The data collected from our Excel tracking is used to drive the focus for community education and outreach on cancer screening and prevention. We also utilize the data to ensure that we are meeting national guidelines for our cancer care and screenings.” (Pilot Site B) “they could come here for their chemo and radiation but we lose them for surgery. And I think that’s important information to know… you start to drill down and figure out why you are losing your surgical patients” (Pilot Site E)
Reporting	“We provide general program information to our Board of Directors as well as to our donors in quarterly and annual reports to show how the program is being accessed by the patient community and the ways in which we are serving them.” (Pilot Site F) “We are also experiencing a turnover in Administration which will require us to provide more specific numbers for navigation services and the downstream revenue we generate since our navigation program is provided as a complementary service for our patients.” (Pilot Site B) “We are hoping to get an oncology social worker, so…being able to…show people how much time is spent on the navigation side of things doing that psychosocial piece…is going to be very beneficial for us.” (Pilot Site B)
Research	“It will better if we have…universal data to track information for research.” (PN, Phase I Survey) “This could be an extremely helpful way to make navigation more uniform and to be collecting the same types of data and things like that across the country and I think it’s just really incredible” (Pilot Site B) “The research side of our organization would love to be able to find a way to take the treatment information that’s collected and report on that” (Pilot Site F)
PN information systems considerations	
Degree of burden on navigators/facilitation of navigation work	“Many nurse navigators have no clerical support nationally, resulting in them spending time doing clerical tasks.” (Admin, Phase I Survey) “So let us just say on follow up dates, for example. If you could sort by follow up date, you could come in every day and have your to do list for the day.” (Pilot Site E) “Should be able to use the EMR to document and retrieve information one is wanting to follow - easily retrieveable [sic].” (Admin, Phase I Survey)
Ability to meet diverse needs	“There are many types of navigation and finding a solution that fits all can be difficult. However, I’m glad to see that attempts are being made.” (Admin, Phase I Survey)
Affordability	“Many institutions are not going to invest in a software specifically for navigation - especially if they have made huge investments in an EPIC type platform. We’ve got to find ways that these large EHR’s can provide the functionality we need and then vendors need to work to share the best practices that their customers come up with!” (Admin, Phase I Survey)
Integration across systems	“best use would be navigation-specific software that would communicate with EPIC< ARIA and VARIAN.” (Admin, Phase I Survey) “We struggle with this as we do not have an electronic system specific for navigation and our hospital system uses multiple systems for various services. Also, we work with private practices that each have their own separate systems, which our navigators do not have access. This makes gathering medical information for patients very difficult and time consuming.” (Admin, Phase I Survey)
Ability to run reports	“Right now we are kind of just doing free flow text notes into our EMR system, when we have a patient or do something. And I think that that’s not showing any [patient] volume really.” (Pilot Site B) “We currently document metrics on flowsheets in Epic. We find this cumbersome and not always useful to the clinical staff. Also, obtaining reports that are meaningful is still a work in progress.” (NN, Phase I Survey) “The ability to document appropriate care, quantify it, and spit out forms for statistics, patient care & education.” (NN, Phase I Survey)
Ability to share data	“Having a seamless referral mechanism to nurse navigation in Epic would be great.” (NN, Phase I Survey) “I may make a referral to dietary, I may make a referral to palliative care, I may make a referral to a social worker, I may have to get them transportation. And those are all things that I would just also go in turn and document in the EMR so that not only I can be the only one to see it, but that it becomes part of their permanent record if someone calls and said, ‘well you know they never offered me transportation assistance’ so I document all that so that it’s there, so I did it.” (Pilot Site A)

*“Admin” was defined as having a supervisor, program manager, coordinator, or evaluator role for a navigation program; *NN*, nurse navigator; *PN*, patient navigator (non-clinically licensed)

### PN information systems landscape

#### Diverse data systems, simultaneous use

Among Phase I survey respondents, Microsoft Excel spreadsheets (46.94%), paper forms (39.36%), and limited use of EHRs (37.03%) were the most common tools used for data collection. A large assortment of EHR, case management, contact management, and office productivity platforms were named, including Epic, Varian, MOSAIQ, Outlook, Word, Salesforce, NeonCRM, Cordata, and Nursenav. Over half (60.35%) reported more than one data collection method.

#### Tailoring issues

While a minority of PN programs reported use of navigation software, typically, information management systems were not specifically designed to meet the needs of PN programs. As Pilot Site B described, “Our organization uses Meditech. Its Oncology Module does not have a Navigation component, which requires us to utilize an outside tracking program.” Pilot Site E noted, “Our process is very disjointed, but the staff find a way somehow to make it work.”

#### Data systems decisions in flux

Several respondents mentioned transitioning between data management systems and experimenting to find better solutions. Pilot Site C described system shopping: “our team is new, just hired my second RN so we are in the process of establishing metrics, trying to identify the best software to use. How do we collect data that is time and energy efficient?” Those operating within larger organizations were also downstream of organization-wide information system changes.

### PN data uses

Navigation programs tracked a wide variety of variables to fulfill diverse needs. For example, an administrator at Pilot Site E described at least seven ways that data was used for her program ranging from tracking patient screening compliance, to providing appointment assistance, to contributing to state evaluation and progress reports.

#### Direct provision of navigation services

Navigators used data to complete day-to-day tasks addressing patient needs: “I keep notes about time and place and subjects discussed as it help[s] me track how patient is doing throughout the process” (Volunteer/ peer navigator, Phase I survey). Many tracked contact information, cancer care details, or appointment dates to expedite services and remind patients of appointments. Navigators with a clinical scope tracked details to generate survivorship care plans and treatment summaries.

#### Service and care improvements

Navigation staff recognized the usefulness of data in evaluating performance, identifying gaps in care, and informing improvements to navigation or cancer center services. For example, an Oncology Director at Pilot Site H suggested that data revealing high external referral rates to meet a specific type of patient need could make the case for “trying to get that service here closer to home.” Navigation staff also wanted to identify outmigration trends to improve patient retention, track performance against national guidelines, and track care dates to identify delays in care.

#### Reporting

With an eye towards growth and sustainability, navigation programs needed data to demonstrate the value of their work to others and justify allocation of resources: “we were able to get a second nurse navigator because we were able to provide a lot of evidence…as to why that was necessary…however my task is to go back at the end of June [with]…metrics that show that this truly was something that was beneficial to the Cancer program” (Pilot Site C). Time tracking emerged as an important economic variable often unavailable in current systems. As a social worker summarized in the Phase I survey: “The institution is always looking at finances as the bottom line. Does the navigator make the hospital money or prevent them from losing money[?]” PN programs also expressed interest in supporting Commission on Cancer (CoC) accreditation and other external reporting.

#### Research

PN data’s importance in contributing to generalizable knowledge was recognized. A few also acknowledged the need for standardization and uniformity in navigation metrics and data collection to help drive the PN field forward.

### PN information systems considerations

Several information systems characteristics impacted attractiveness and feasibility of adoption to PN programs, described below.

#### Degree of burden on navigators/facilitation of navigation work

The burden of collecting, entering, and managing data emerged as a substantial concern for navigation staff who were often tasked with these responsibilities in addition to patient care. As a nurse navigator expressed in the Phase I survey, “...documenting value of navigation requires data/statistics which is an administrative need, not a patient need, and not my need…don’t take away precious time patients/families need from a navigator by having the focus on statistics.” Navigators lacked time and clerical support, varied in technological literacy and comfort, and strongly believed in prioritizing patient care over data-related tasks. An administrator highlighted the importance of end-user perceived usefulness in promoting quality data collection and management: **“**Because documentation is a lot of work and for navigation, falls on individuals that are hard-pressed to find sufficient time for everything else, any documentation systems have to be of immediate as well as long-term value as perceived by the navigator. If not, it will either not get done, or not get done promptly and well” (Phase I survey). Data systems able to easily retrieve client information and provide appointment reminders, task lists, or automatic referrals were described as useful. PNs valued streamlining, user-friendliness, avoidance of double documentation, and time-saving features**.**

#### Ability to meet diverse needs

PN-BOT™ pilot testing revealed the impossibility of defining a single set of variables both adequately detailed and universally applicable across diverse navigation contexts. For example, race/ethnicity data was important for Pilot Site G, which needed to demonstrate ability to reach specific minority patient populations for grant application purposes. Other programs were uninterested in this variable. Navigators did not want to see irrelevant variable fields; yet, specialized navigators wanted entry fields that did not apply to all patients. For instance, breast navigators wanted breast imaging reporting and data system (BI-RADS) categories and those in surgical settings wanted pathologic stage. Thus, it was important for information systems to be customizable to meet different PN needs.

There were also competing priorities between PNs and other stakeholders: “my clinic recently went to a new charting system and it looked like it would be great to use from the I.T. department’s point of view, but no one bothered to see what the end user (nursing, physician & clerical staff) thought. The new system is not giving us the measurements that we need to see and measure in regards to patient navigation” (Nurse navigator, Phase I survey). Navigators lacked agency in some cases to make data-related decisions in their settings.

#### Affordability

Navigation staff referenced the high cost of software designed specifically for navigators. Pilot Site G explained, “We were considering purchasing a new software program. In researching them, we found most are too expensive and designed for hospitals. We are a small non-profit organization.” A hospital-based Phase I survey respondent also referenced cost as a consideration, especially in light of recent organizational investments in expensive EHR systems for non-navigation purposes.

#### Integration across systems

Navigation staff expressed frustration at a fragmented landscape involving use of multiple siloed systems: “EMRs need to be more integrated. At many of our clinics/hospitals each specialty/clinic has their own EMR that does not ‘talk’ to any of the other EMRs. This makes it extremely difficult for our navigators to identify which services patients have had and next to impossible to produce accurate screening rates from the EMR” (Administrator, Phase I survey). Such lack of interoperability increased risk of issues falling through the cracks and created difficulties with data sharing, continuity of care, and duplicate data entry.

#### Ability to run reports

Navigation staff indicated strong interest in the ability to retrieve synthesized reports from information entered and identified shortcomings of information systems currently in use: “although I document in the chart, I am not able to really get an accurate report to prove my worth” (Nurse navigator, Phase I survey). Data collection fields sometimes lacked the structure to provide reports in a sufficiently quantitative or detailed way. Reports sometimes required additional cumbersome work. As a nurse navigator described in an interview, “I tend to use my Excel spreadsheet now, and I hand count things. So to be able to have a system that can generate reports is going to be extremely beneficial to us” (Pilot Site E).

#### Ability to share data

Since navigators often work as part of a medical team with referral relationships, the ability to share data was important. A navigator at Pilot Site E described, “…if I put a note into the electronic medical record where the surgeon, the medical oncologist, they’re on that same system, they can see exactly the date I met with the patient and what I discussed with them. It’s all in there, in their EMR.” Data sharing was important to manage shared patient caseloads and have accountability.

## Discussion

Navigators, tasked with guiding patients through fragmented healthcare systems, also navigate fragmented information systems. PN programs are resourceful when they cannot access ready-made tailored solutions, shopping and experimenting with a patchwork of imperfect information systems. The data suggest that information systems with high perceived usefulness to PN programs should have the ability to facilitate navigation services, service improvements, reporting, and research. Low data entry burden, high accessibility of entered data, customizability, affordability, and interoperability were identified as important characteristics for ease of use. Our finding that PN programs desired but lacked interoperable systems was echoed in past health information technology research [24].

The discovery of diverse data needs was unsurprising, since PN programs arise out of diverse contexts to meet needs specific to their institutions and patient populations. Though PN programs commonly desired information system attributes like low entry burden and streamlined systems, they diverged in specific variables desired, types of reports needed, and resources available. It is an ongoing challenge to balance standardization and rigor in the field with flexibility and responsiveness to individual PN program needs. Shortly after the development of PN-BOT™, the Academy of Oncology Nurse and Patient Navigators (AONN+) convened a task force which proposed 35 evidence-based standardized metrics for use by PN programs to enable comparability across programs [7, 11]. Metrics spoke to business performance, clinical outcomes, and patient experience, with clear definitions to promote consistent operationalization [7, 11]. Others have proposed common cost and patient-reported outcome measures [9, 12]. Such guidance promotes comparable measurement while providing a menu of options for PN programs to choose from to meet their specific evaluation needs. Incorporation of standardized metrics into information systems designed for PN programs, with the capability of adding supplementary context-specific metrics, can further encourage high-quality data collection in the field.

The final PN-BOT™ product was designed to incorporate several characteristics considered important to PN program stakeholders: support of day-to-day tasks, affordability, high accessibility of entered data, ease of reporting, and customizability. PN-BOT™ does not currently address interoperability needs in terms of EHR integration, but uses an Excel platform which is common and familiar in office settings. Data entry burden is contextual based on the number of variables chosen for use within PN-BOT™ and external PN program documentation obligations. A mobile application designed for PNs was recently developed by Rohan and colleagues with promising evaluation findings, reflecting ongoing efforts in the field to find innovative PN data needs solutions [25].

### Limitations

Participants reflected volunteer convenience samples. PN programs with less computer use or connection to professional networks may have different data management needs and experiences. Since the primary purpose of data collection was to inform development of a new, free data management tool, participants may have had especially large unmet data needs, openness to change, and/or strong interest and opinions on PN evaluation and metrics. PN programs more satisfied with their current strategies or less in need of reporting metrics were likely underrepresented. Therefore, findings may not be generalizable to all PN programs.

### Practice implications

PN programs need flexible, rigorous tools for data management and reporting. Information systems designed for PN programs should integrate seamlessly with other systems and offer PN-oriented features, such as contact management, referrals, encryption, information sharing, scheduling and calendar reminders, and report generation. For programs embedded in larger organizations, open communication between PN staff and decision-makers about data needs could improve EHR selection, maximize the utility of existing systems, and reduce inefficiencies. Since high-quality data is unattainable without high-quality data entry, it is important to listen to “front lines” PN staff, value their time as finite, and ensure that adequate supports are in place. If systems are not user-friendly, data entry is not perceived as being immediately useful, or burden is unreasonable, navigation staff will be hard pressed to prioritize and effectively use information systems. Although the demand exists for an information system able to meet specific PN needs, not all programs have resources for expensive solutions. Therefore, affordability is necessary for adoption to be realistic.

Professional organizations and technical assistance providers can support PN programs exploring data options by facilitating dissemination of successful practices and lessons learned among PN programs. Future research can monitor progress as new technologies are developed, and measure the potential impact of such systems on increasing evaluation and research capacity among oncology PN programs.
